# A Belgian View of Medical Advancement

**Published:** 1890-02

**Authors:** 


### A Belgian View of Medical Advancement.

It is pretty generally conceded that too many doctors exist in all portions of the world. As the law forbids killing the surplus, other measures are suggested to remedy the difficulty. In a late issue of the London *Lancet* is given the views of the Belgian Medical Federation. This body affirms that the ranks of the profession are overcrowded because a large number of young men of no particular ability, from the artisan and lower classes, have the idea that the practice of medicine is a nice light sort of work. To stop this it is proposed to require, as a preliminary to entering upon medical study, a very complete classical education, with a good knowledge of modern languages. Thus mediocracy would be shut off at the very beginning. It is also insisted that students of pharmacy should have the same preliminary training, because these individuals trench easily upon the field of the physician.

We think that the Belgians have hit upon the key-note of the entire question. Preliminary training needs to be increased over the entire world. Once secure the general idea that the proper study of medicine, as at present understood, is impossible, except to him who has the equivalent of the degree of A. B., and the profession would no longer be surfeited by too many members. To most medical students the new requirement would mean at least six or eight more years of hard study. Medicine then would not seem the easy, light pursuit that so many now regard it.—*American Lancet.*

